# Circulatory Death and Organ Donation

**DOI:** 10.31729/jnma.8437

**Published:** 2024-02-29

**Authors:** Badri Man Shrestha

**Affiliations:** 1 Sheffield Kidney Institute, Sheffield Teaching Hospitals NHS Trust, Sheffield, United Kingdom

To address the global shortage of organs for transplantation, donors after circulatory death (DCD) are being increasingly utilised as an invaluable source of organs such as kidney, liver, heart, lungs and pancreas. DCDs are critically ill hospitalised patients, who do not fulfil the brain-stem death criteria and withdrawal of life-sustaining treatment (WLST) is considered because of expected futile outcomes of continued treatment.^1^ The diagnosis of circulatory death is confirmed by the permanent absence of systemic circulation, which eventually leads to cessation of brain perfusion and permanent cessation of brain functions. This editorial focuses on the basic science and clinical evidence pertinent to the strategies adopted to enhance the utilisation and outcomes of kidney transplantation (KT) from DCD donors.

The impact of associated pre-morbid conditions in the donors and extended warm and cold-ischaemia times (CIT) during and after organ recovery leading to higher incidence of delayed graft function (DGF), primary nonfunction (PNF) and compromised long-term outcomes, has been the subject of concern in the past. With an enhanced understanding of the pathophysiology of the DCD and experience gained over the past three decades, KT from DCD donors has become routine.^2^ In recent years, a marked increase in DCD donation rate has occurred in Spain (19.55 per million population (pmp), Belgium (12.82 pmp), Netherlands (10.02 pmp), United Kingdom (9.43 pmp), France (3.58 pmp) and China (1.29 pmp).^3^

Formerly known as non-heart-beating, asystolic or donors after cardiac death, the concept of DCD was introduced by Kootstra et al. in Maastricht, Netherlands.^4^ These donors are classified as uncontrolled donors (Category 1: brought in dead; Category 2: unsuccessful resuscitation; and Category 5: cardiac arrest in a hospitalised patient; or controlled donors (Category 3: awaiting cardiac arrest; and Category 4: cardiac arrest after brain-stem death).^5^ Countries like the UK and Australia focus principally on controlled DCD, whereas France, Spain and the Netherlands utilise both uncontrolled and controlled DCD donors.

The process of DCD donation includes the completion of a checklist for organ donation followed by WLST in the operation theatre and awaiting circulatory arrest (asystole) to occur. Before death is confirmed, five minutes of observation is carried out after the asystole to confirm irreversible cardio-respiratory arrest, which is confirmed by the isoelectric line in the electrocardiogram tracing and the absence of blood pressure in the arterial line. The time allowed from WLST to asystole is accepted as 2 hours for the kidney and 1 hour for the liver and pancreas. This is extended to a further 2 hours for the kidney, and 30 minutes for the liver and pancreas based on the functional warm ischaemia, which starts when the blood pressure falls below 50 mm of Hg and oxygen saturation is below 70%. Otherwise, the team stands down the retrieval process.^5^

A report from the UK transplant registry showed DGF in1882 of 4714 KT (39.9%) from controlled DCD donors to adult recipients. After risk adjustment, the presence of DGF was not associated with inferior long-term graft or patient survivals. However, a DGF duration of >14 days was associated with an increased risk of death-censored graft failure (hazard ratio 1.7, p = .001) and recipient death (hazard ratio 1.8, p < .001) compared to grafts with immediate function.^6^ There was no difference in the 3-year patient (91.4 vs. 92.2%) and graft (88.2 vs. 90.0%) survivals between DCD and DBD kidneys. By far, kidneys from donors aged >60 years had more than twice the risk of graft failure within 3 years of KT compared with those transplanted with kidneys from donors <40 years (HR 2.35, p<0.0001).^7^

The optimum method of preservation of DCD kidney using static cold storage (SCS) or hypothermic machine perfusion (HMP) was assessed in an international randomised controlled trial from Europe, which did not show a significant difference in the incidence of DGF between the two methods of storage.^8^ Follow-up of the recipients from the same study did not show a significant difference in the 3-year graft survival between the two methods of storage.^9^ A multicentre randomised trial conducted in the UK for DCD kidneys, showed no difference in the incidence of DGF (58% vs. 56%), renal function at 3 and 12 months, graft and patient survivals; thus concluding HMP offered no advantage over SCS and the latter was cheaper and more straightforward.^10^ Currently, SCS is used by all transplant centres in the UK for organ preservation.

Normothermic regional perfusion (NRP) is an in-situ perfusion strategy to reperfuse organs with oxygenated blood and reduce warm ischaemic damage, which is gaining popularity. Application of NRP in DCD donors in the UK showed a significantly increased rate of utilisation of kidney (1.5-fold), liver (5-fold) and pancreas (1.6-fold), reduction of DGF and improved transplant outcomes in the NRP group compared to conventional organ recovery.^11^

Implantation of two kidneys in one recipient (dual kidney transplantation) from adult DCD and other extended criteria donors, which otherwise might be discarded for functional reasons, is being increasingly practised. Excellent graft and patient survival outcomes and a reduction in waiting time have been achieved, particularly in the older recipient population.^12^

To establish a DCD programme, it is important to develop a protocol by a team of hospital personnel including intensivists, surgeons, nephrologists and the legal department and maintain transparency to ensure public trust in the system of organ donation. Initially, the adoption of a conservative approach by accepting a selected group of controlled young DCD donors with preserved organ function and allocation to appropriate recipients is essential. Maintenance of a robust database, regular audit of the outcomes and extension of donation criteria with broadening of experience, are essential for establishment of a successful DCD programme in any institution.
